# Different approaches to managing bilateral distal femur periprosthetic fractures in a single patient: case reports and review of the literature

**DOI:** 10.1093/jscr/rjae767

**Published:** 2025-02-10

**Authors:** Christopher Castagno, Kyle J Klahs, Adam Adler, Mehrdad Hosseini, Brendon Ofori, Amr Abdelgawad, Ahmed M Thabet

**Affiliations:** Texas Tech University Health Science Center, Paul L. Foster School of Medicine, El Paso, TX 79905, United States; Department of General Surgery, Marshall University, Huntington, WV 25702, United States; Maimonides Medical Center, Brooklyn, NY 11024, United States; Texas Tech University Health Science Center, Paul L. Foster School of Medicine, El Paso, TX 79905, United States; Texas Tech University Health Science Center, Paul L. Foster School of Medicine, El Paso, TX 79905, United States; Texas Tech University Health Science Center, Paul L. Foster School of Medicine, El Paso, TX 79905, United States; Maimonides Medical Center, Brooklyn, NY 11024, United States; Texas Tech University Health Science Center, Paul L. Foster School of Medicine, El Paso, TX 79905, United States

**Keywords:** total knee arthroplasty, distal femur periprosthetic fractures, elderly, retrograde intramedullary nail fixation, distal femur replacement, knee injury and osteoarthritis outcome score

## Abstract

A 61-year-old female with a bilateral total knee arthroplasty (2010) and subsequent separate bilateral distal femur periprosthetic fractures (2020 and 2022) treated with distal femur replacement (left) and retrograde intramedullary nail fixation (right). Self-reported outcomes and knee injury and osteoarthritis outcome scores (KOOS) were compared. The patient reported higher pain and lower functionality with the left knee (overall KOOS of 50%) and no lasting impact or pain with the right knee (overall KOOS of 89%). When KOOS individual (*P*-value of <0.0001) and categorical/overall (*P*-value of 0.0048) metrics were compared they demonstrated statistical significance. The report’s data, based solely on a one-sample-size data set, suggest that performing an intramedullary nail for distal femur periprosthetic fractures should be the preferred choice based on this patient’s improved pain, functionality, and overall satisfaction, even if anatomic alignment is difficult.

## Introduction

Distal femur (DF) fractures comprise ~1% of all fractures and 4–6% of femoral fractures. The incidence of these fractures is increasing due to an aging population [1–4]. The treatment options for DF fractures have evolved in recent years. There are two main surgical categories of treatment: fixation and arthroplasty. Fixation methods include plating, intramedullary nailing, and nail/plate combination. The specific treatment option depends on the nature of the fracture and the individual circumstances of the patient [4–9].

Recently, there has been growing interest in comparing the outcomes of distal femoral replacement (DFR) and open reduction internal fixation (ORIF) for these fractures. DFR is designed to provide early weight-bearing and range of motion (ROM), and ORIF, on the other hand, allows the patient to maintain their native bone stock. As of the time of this publication, no published randomized controlled trials have thoroughly compared these two treatment modalities. KOOS scores were analyzed using GraphPad Prism and statistics based off a paired T-Test. This unique case series provides both an objective comparison, using the knee injury and osteoarthritis outcome score (KOOS) (Table 1) and pain scores reported using the standardized visual analogue scale, and a subjective comparison, based on perceived ROM, balance, functionality, and a few general novel questions (Table 2) comparing these different surgical interventions [10]. KOOS individual question scores, categorical percentages, and overall category percentages were statistically analyzed using GraphPad Prism based off a paired T-Test.

**Table 1:** Template, KOOS.

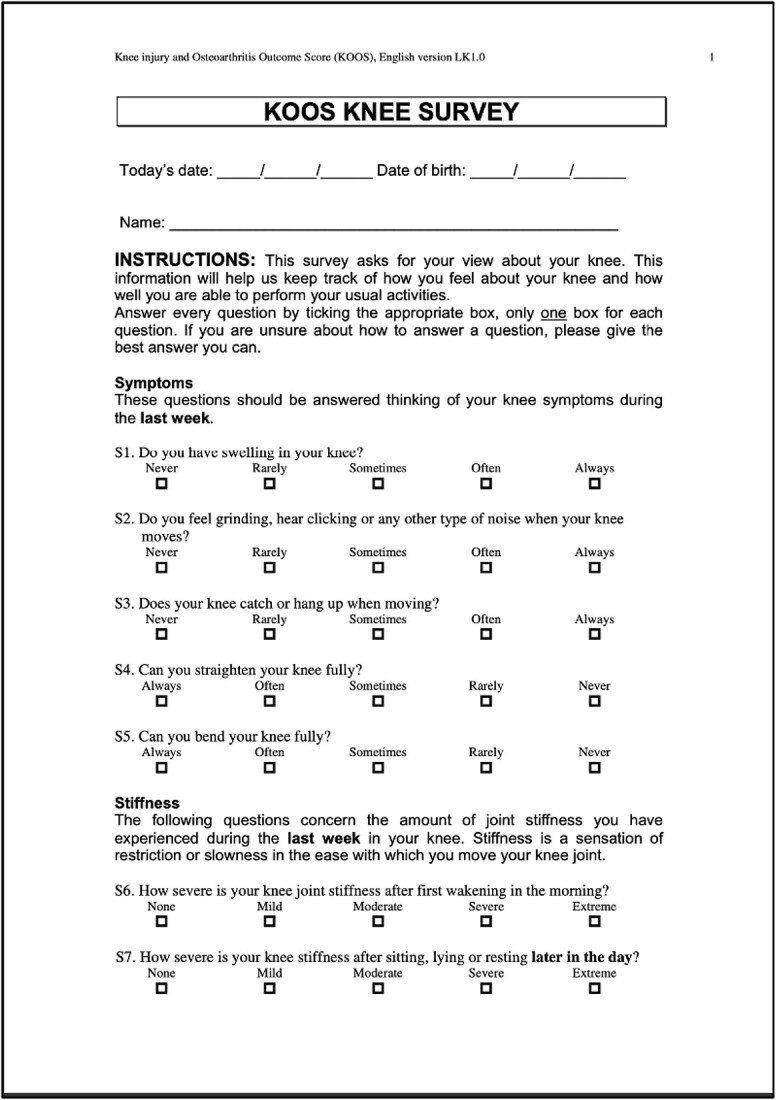
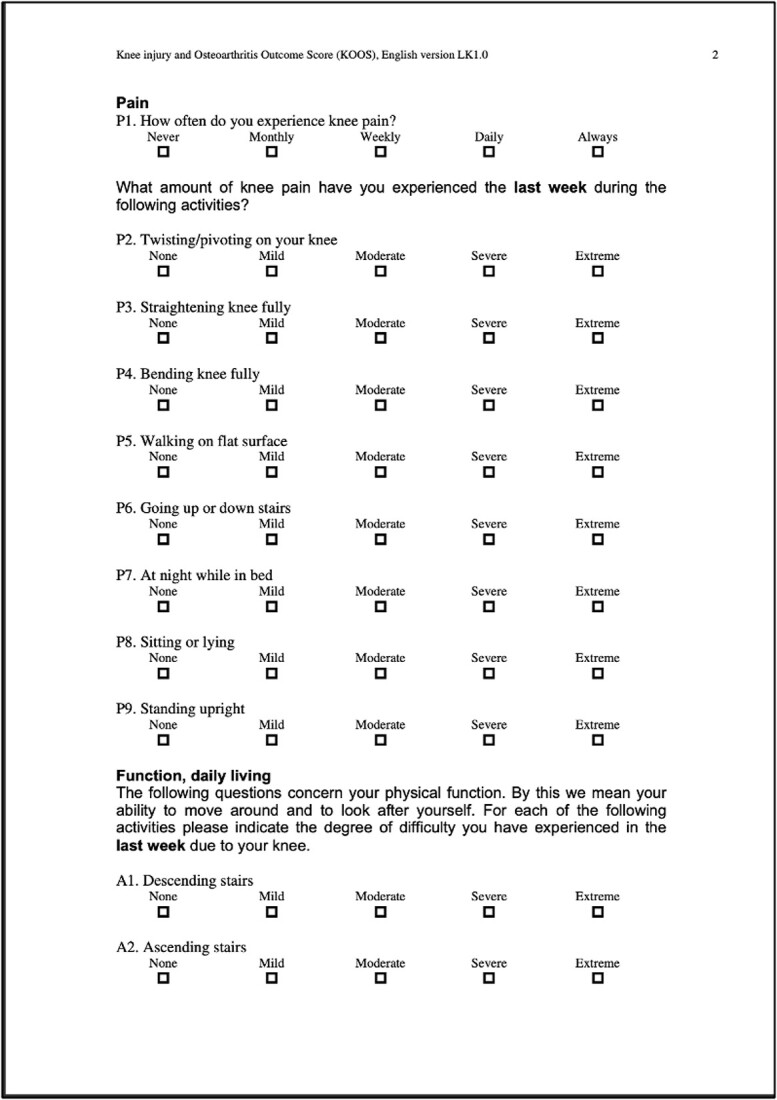
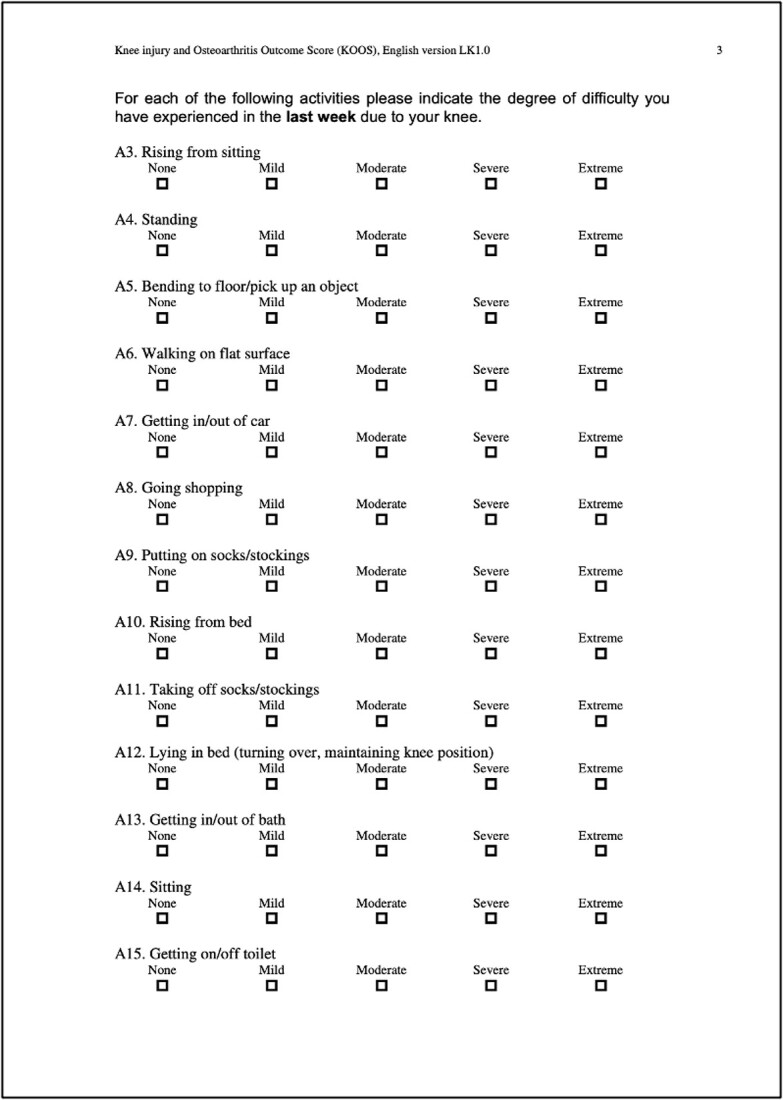
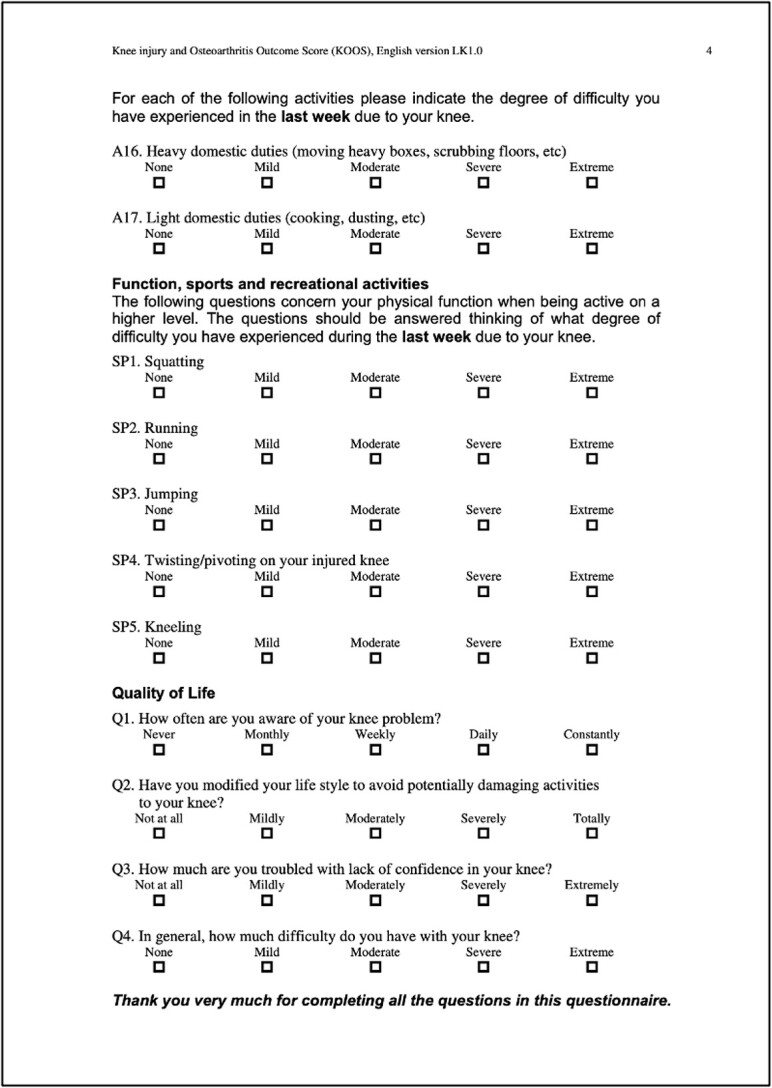

**Table 2:** Reported Outcome Score. General novel questions asked to the patient.

**General Questionnaire about Postoperative Outcomes and Differences**
Are there any differences in the recovery process between the two knees? Have you noticed any difference in pain levels between the two knees since the surgery? Do you have more or less range of motion in one knee compared to the other? Have you experienced any complications specific to one knee or the other? Have you been able to walk or stand for longer periods of time since the surgery? Have you been able to resume any activities that you were unable to do before each surgery? Have you noticed any differences in the sensation or feeling in the knees since the surgery? Are there any specific concerns or issues you have experienced related to the surgeries or the recovery process? Are you satisfied with the outcomes of both surgeries? If not, what would you like to improve or address further?

## Case description

### Case 1: Left knee

#### Mechanism of injury and preoperative planning

**Figure 1:**
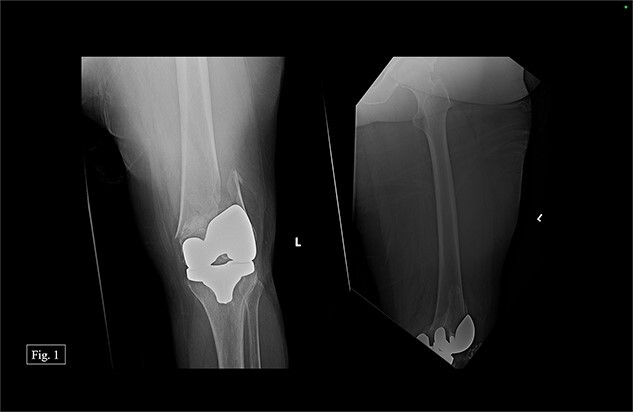
Left, acute distal periprosthetic femur fracture X-ray.

In February 2020, the patient presented with a left distal periprosthetic femur fracture (Su Type II) after attempting to walk to the bathroom and falling onto her left knee (Fig. 1). Due to minimal bone availability for distal fixation around the prosthesis, the patient was indicated for a DFR, allowing immediate weight bearing.

#### Intra-operative

**Figure 2:**
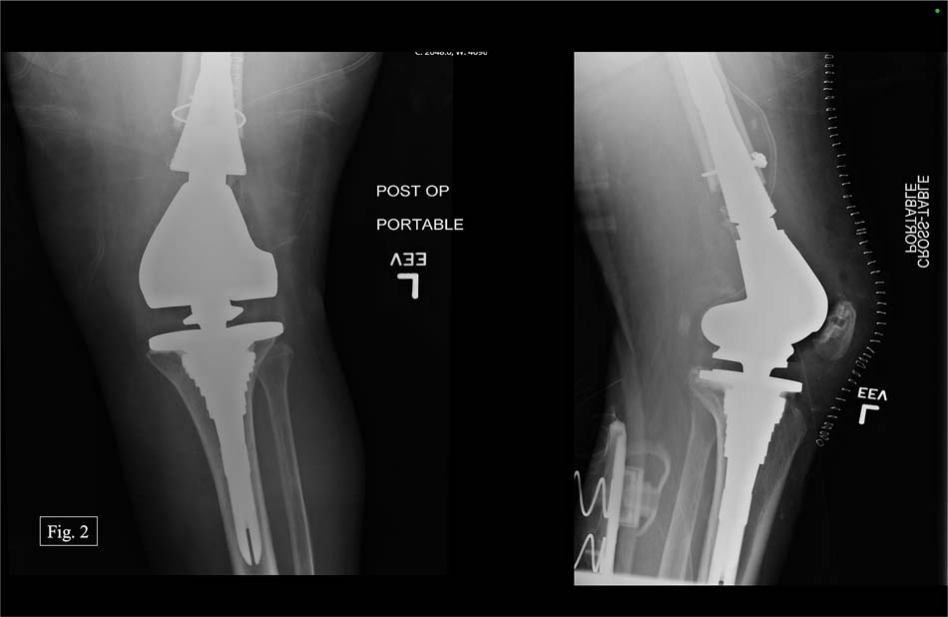
Left, immediate postoperative AP and lateral X-rays.

The previous medial parapatellar approach was used to identify and shell out the fractured DF with an arthroplasty component end block. Press fit trials were used, and the contralateral knee joint height was compared. We used the DePuy Synthes MBT revision metaphyseal sleeve, tibial tray rotating platform, MBT revision cemented, limb preservation system (LPS) tibial insert hinge, LPS distal femoral component, LPS sleeve adapter to distal femoral component, universal femoral sleeve, and a universal fluted stem. The patient experienced 1000 mL of intraoperative blood loss, awoke without incident, and was admitted to the ICU for monitoring and management. Postoperative anterior–posterior (AP) and lateral X-rays were taken (Fig. 2).

#### Clinical course

**Figure 3:**
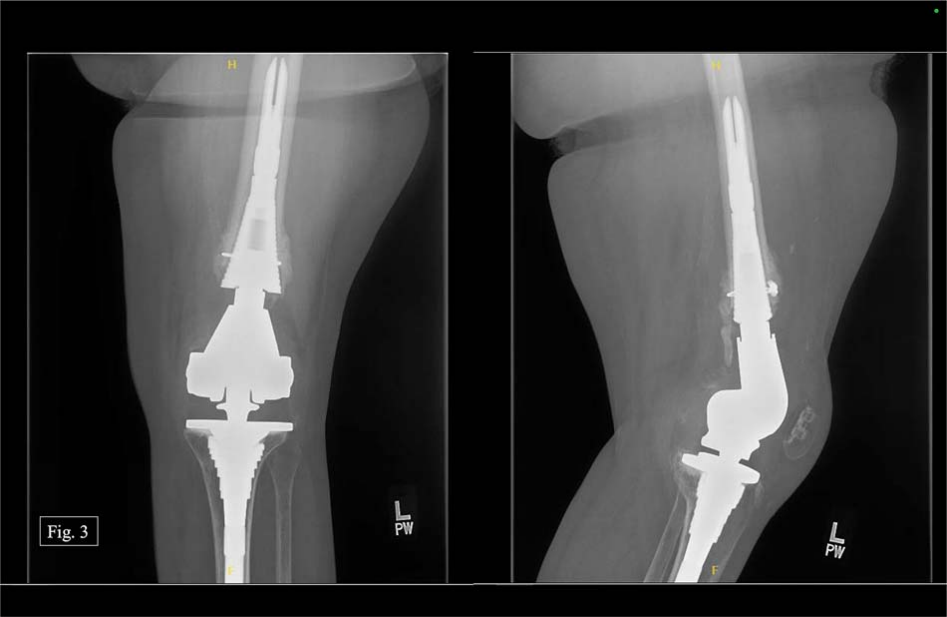
Left, 5 month postoperative AP and lateral X-rays.

After being discharged from the University Medical Center, the patient visited Texas Tech Orthopedic Clinic 2 weeks after the operation. In July 2020, around 5 months after the operation, she stated that she was regularly and consistently doing physical therapy and reported a pain score of 4/10. During the visit, APand lateral left knee X-rays were taken (Fig. 3).

**Figure 4:**
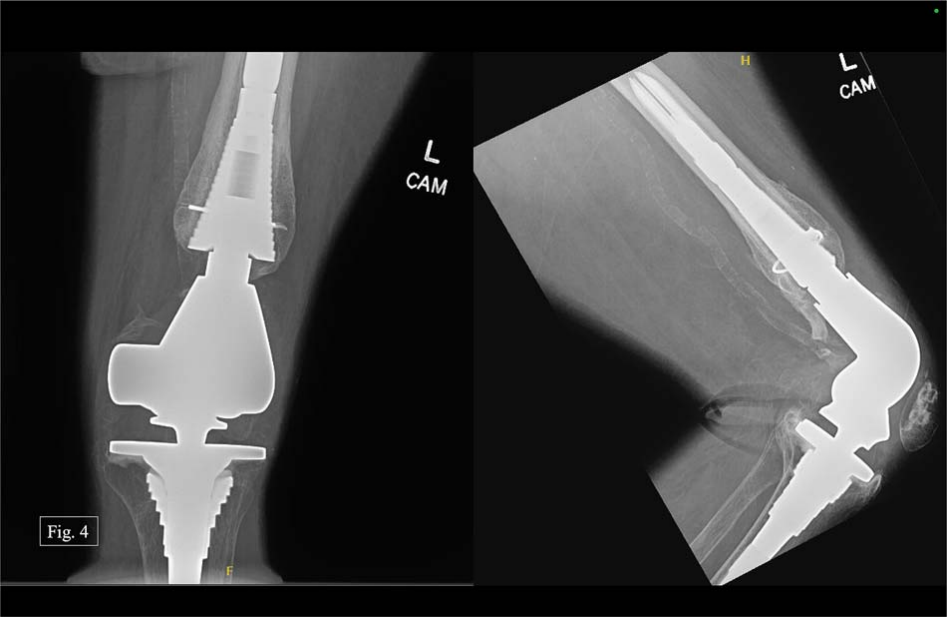
Left, 48 month postoperative AP and lateral X-rays.

During her most recent visit in February 2024, roughly 4 years after DFR, the patient reported a pain score of 5/10 pain at rest, and bilateral AP, right & left lateral, right & left AP, and full leg-length X-rays were taken. Looking at the APand lateral imaging of the left knee, there were findings of DFR without signs of lucency or subsidence, and the hardware-maintained alignment was intact (Fig. 4). She was able to ambulate with moderate, 6/10 pain and had full passive ROM with similar pain. She was instructed to continue at-home physical therapy and non-steroidal anti-inflammatory drugs (NSAIDs) as needed for pain and to return to the clinic in 6 months for further evaluation.

### Case 2: Right knee

#### Mechanism of injury and preoperative planning

**Figure 5:**
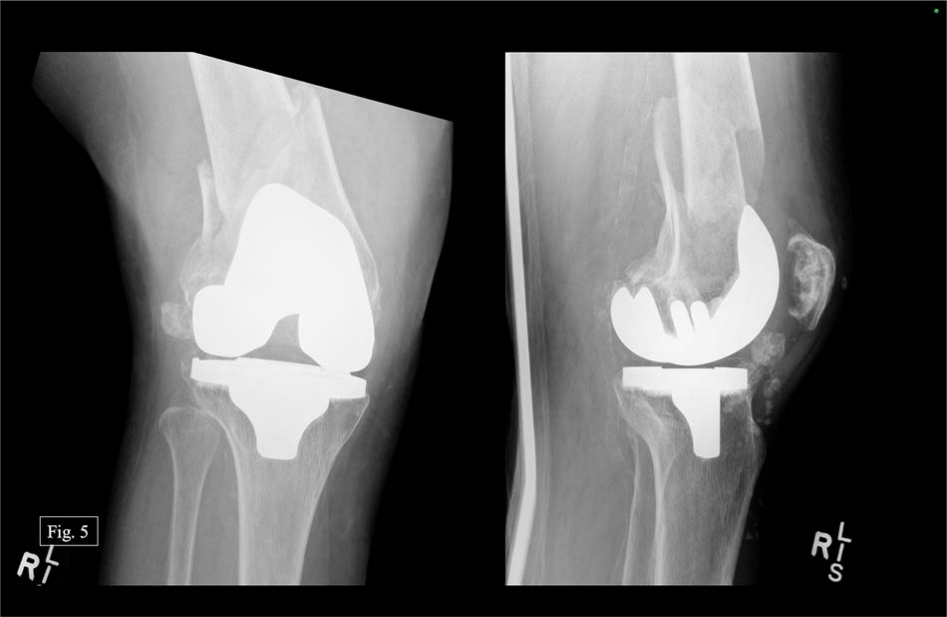
Right, acute distal periprosthetic femur fracture X-rays.

The patient presented to our level 1 trauma center in September 2022 with acute right knee pain and unable to ambulate. She was seen and evaluated in the emergency department, where she was found to have evidence of a right distal periprosthetic femur fracture (Su Type II) after a ground-level fall (Fig. 5). Because of the open box configuration of the native TKA implant and adequate available bone stock, a standard Stryker right retrograde intramedullary nail (IMN) was selected.

#### Intra-operative

**Figure 6:**
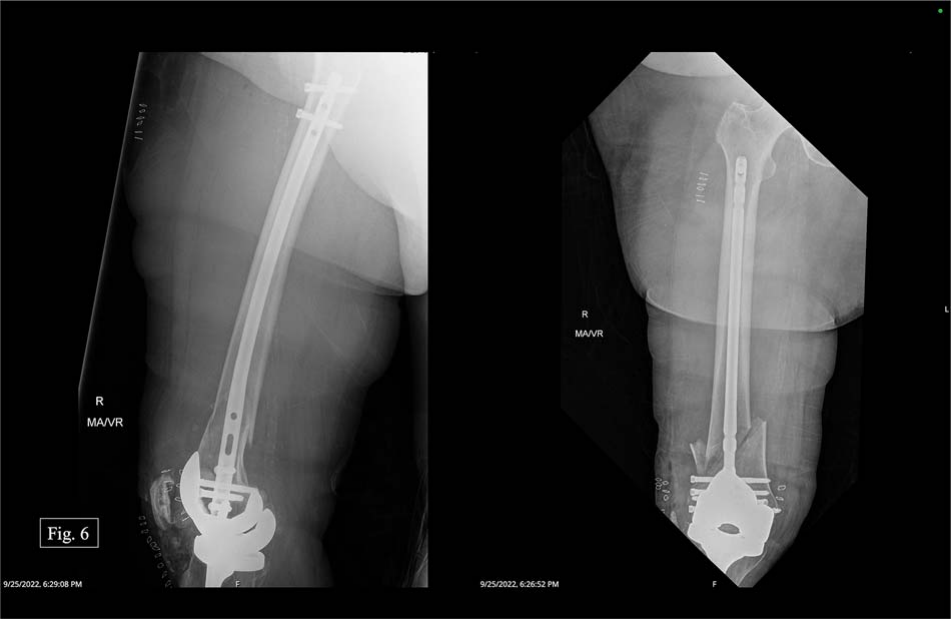
Right, immediate postoperative AP and lateral X-rays.

The patient was positioned supine with a radiolucent triangle under the operative knee. A trans-patellar approach was made for our T2 alpha retrograde nail measuring 10 × 330 mm. We confirmed the appropriate purchase with fluoroscopy and placed the distal interlocking screws through the implant. The distal articular block was noted to be slightly valgus; however, it appeared appropriate when assessing her mechanical and clinical alignment. The patient experienced 100 mL of intraoperative blood loss, awoke without incident, and was admitted to the ICU for monitoring and management. Postoperative APand lateral X-rays were taken (Fig. 6).

#### Clinical course

**Figure 7:**
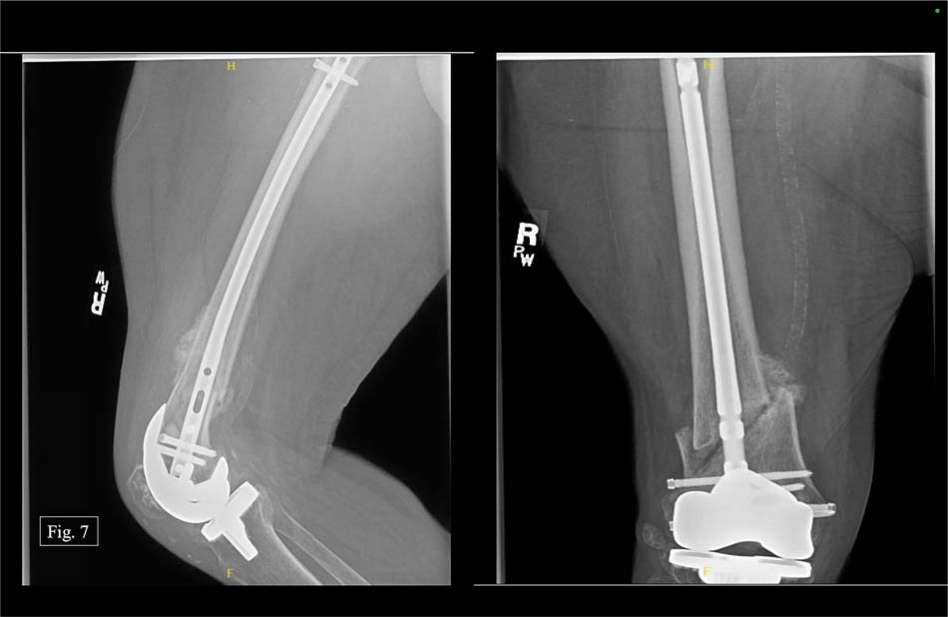
Right, 3 month postoperative AP and lateral X-rays.

The patient was seen at Texas Tech Orthopedic Clinic 2 weeks after discharge from University Medical Center with no postoperative complications. In December 2022, 3 months after being treated on the right with a retrograde nail, the patient reported bilateral knee pain (5/10 on the left and 6/10 on the right). During the visit, AP and lateral right knee X-rays were taken (Fig. 7). She could ambulate with a walker during the visit and reported doing consistent physical therapy at home but no longer seeing a physical therapist.

**Figure 8:**
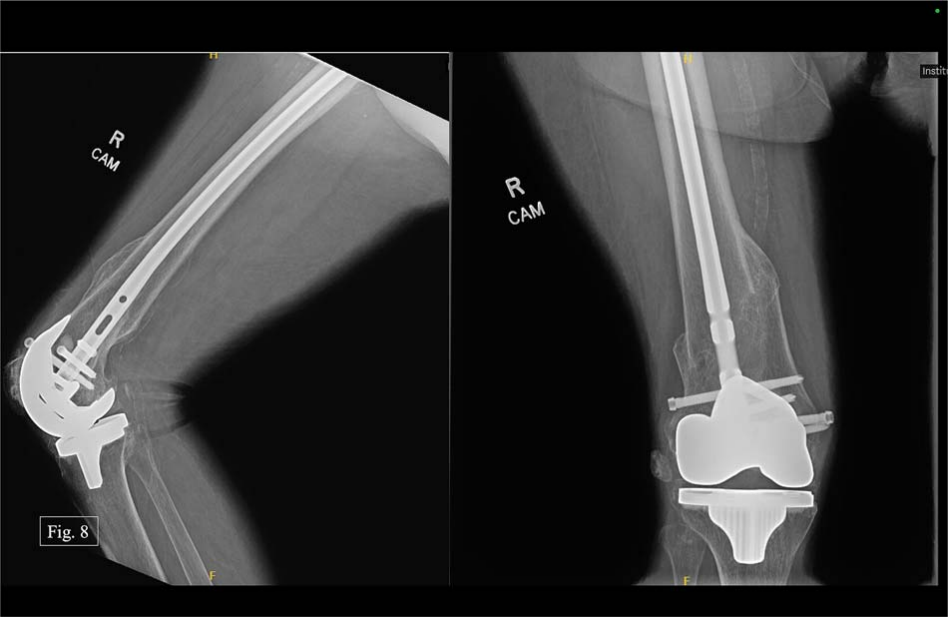
Right, 17 month postoperative AP and lateral X-rays.

**Figure 9:**
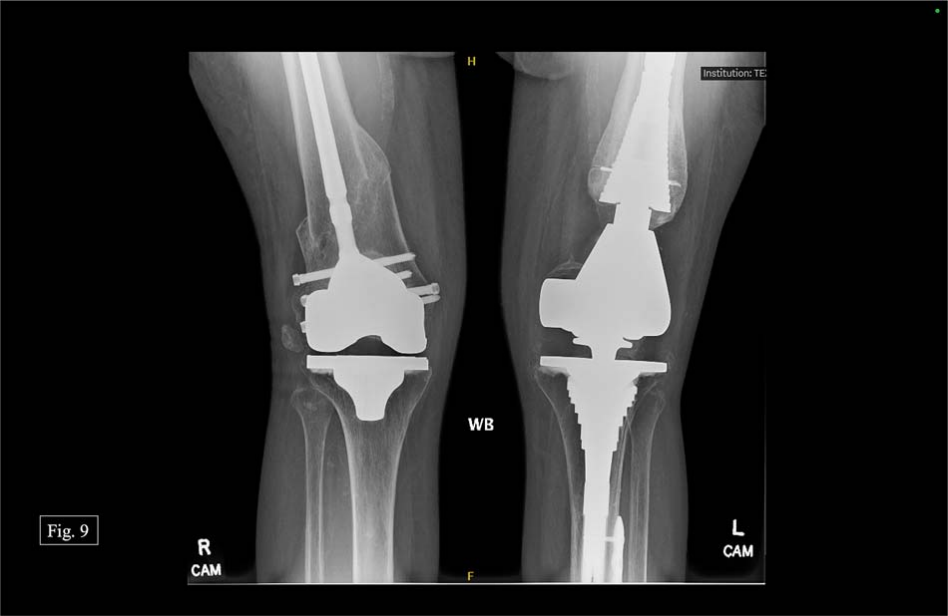
Bilateral, February 2024, AP X-ray.

**Figure 10:**
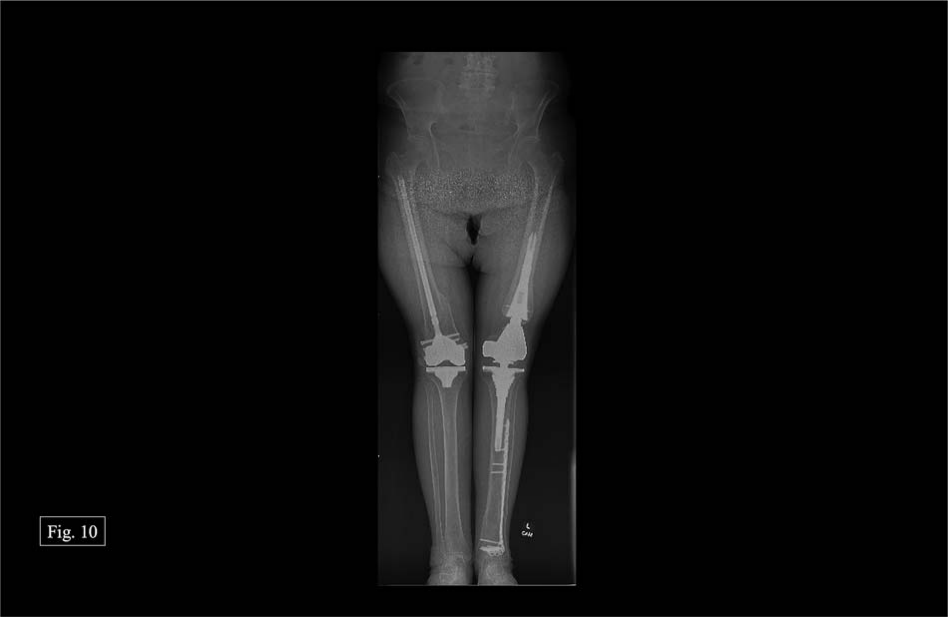
Bilateral, February 2024, full leg length X-ray.

During her most recent visit in February 2024, 17 months post-op on the right, bilateral AP, right & left lateral, right & left AP, and full leg-length X-rays were taken. When looking at the right AP and lateral imaging, she had findings of progressive bone healing, maintained surgical alignment, and all hardware was intact (Fig. 8). Further, when looking at the bilateral AP and full leg length imaging, she had maintained hip alignment, equal leg length, and no changes in valgus or varus leg alignment (Figs 9 and 10). The patient reported a resting right knee pain of 1/10 and an ambulatory right knee pain of 2/10 with a full ROM. She was instructed to continue at-home physical therapy and NSAIDs as needed for pain and to return to the clinic in 6 months for further evaluation.

## Outcomes

### Table 2 presented novel questions to the patient

The patient reports (Table 2) that her left knee has been the most troublesome regarding recovery and pain levels. She states that the left knee primarily affects her ROM and walking ability. Although she has not experienced any significant complications, her injury has had an impact on her quality of life. She can only walk for 1–2 min and requires support to stand due to feeling unsteady. She is unable to carry heavy loads. The patient attributes feeling “off-balance” to the weakness in her left knee. Additionally, she experiences tingling and numbness in her left knee but has no sensation deficits in her right. Although she is content with the outcomes of both surgeries, she desires less pain in her left knee and wants to increase her activity level/walking duration.

### Left-KOOS

Overall score of 50%; (symptoms + stiffness subtotal: 79%; pain subtotal: 58%; function, daily living subtotal: 71%; function, sports, and recreational activities subtotal: 20%; quality of life subtotal: 25%) (Table 3).

**Table 3:** Left, KOOS.

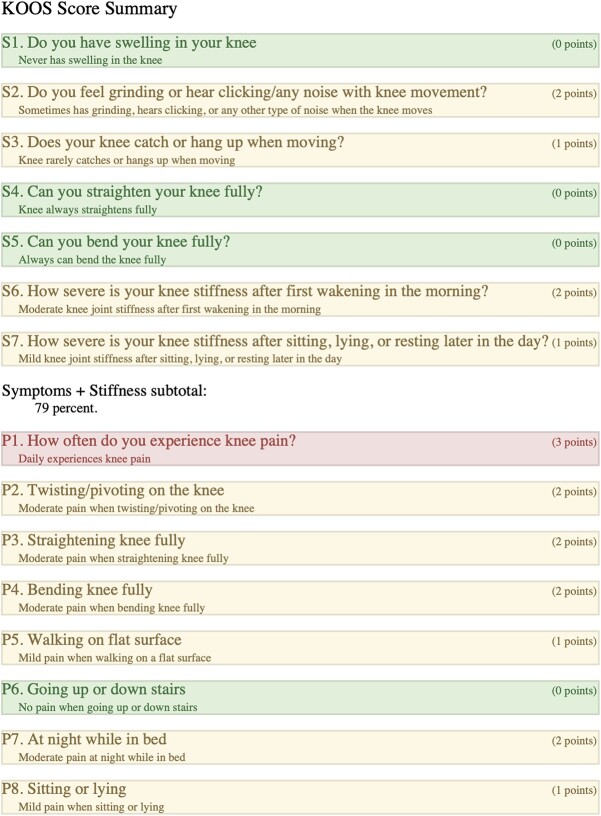
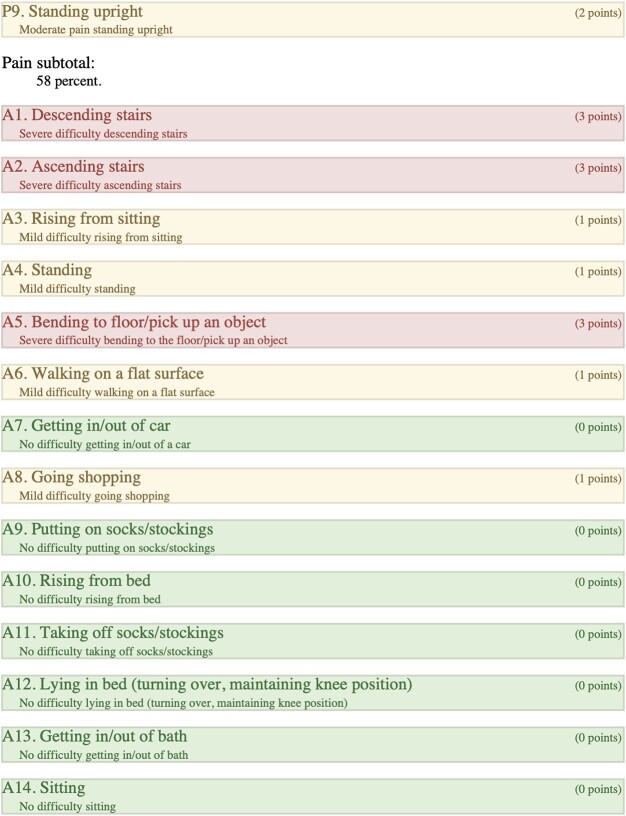
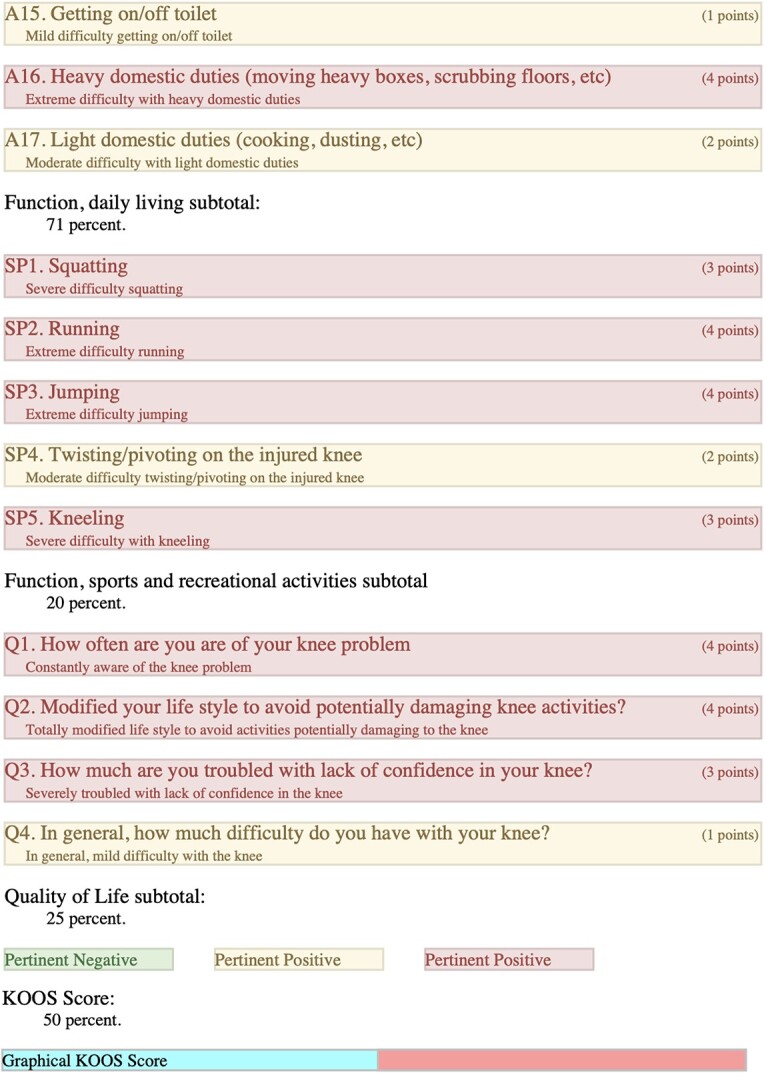

### Right-KOOS

Overall score of 89%; (symptoms + stiffness subtotal: 100%; pain subtotal: 100%; function, daily living subtotal: 100%; function, sports, and recreational activities subtotal: 45%; quality of life subtotal: 100%) (Table 4).

**Table 4:** Right, KOOS.

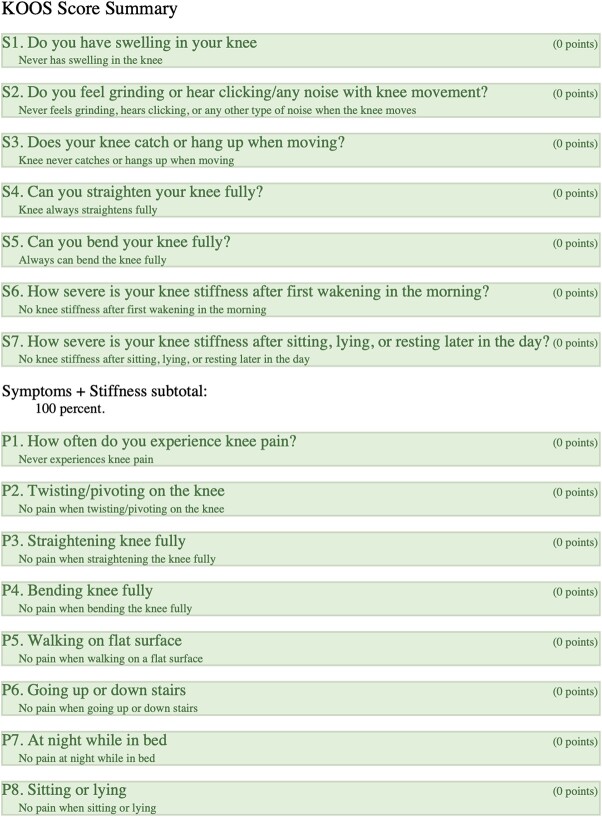
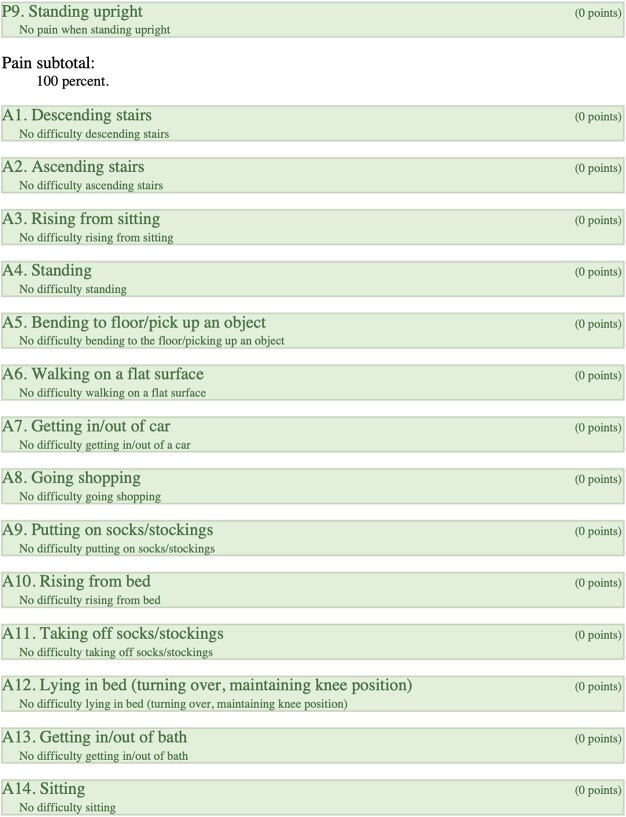
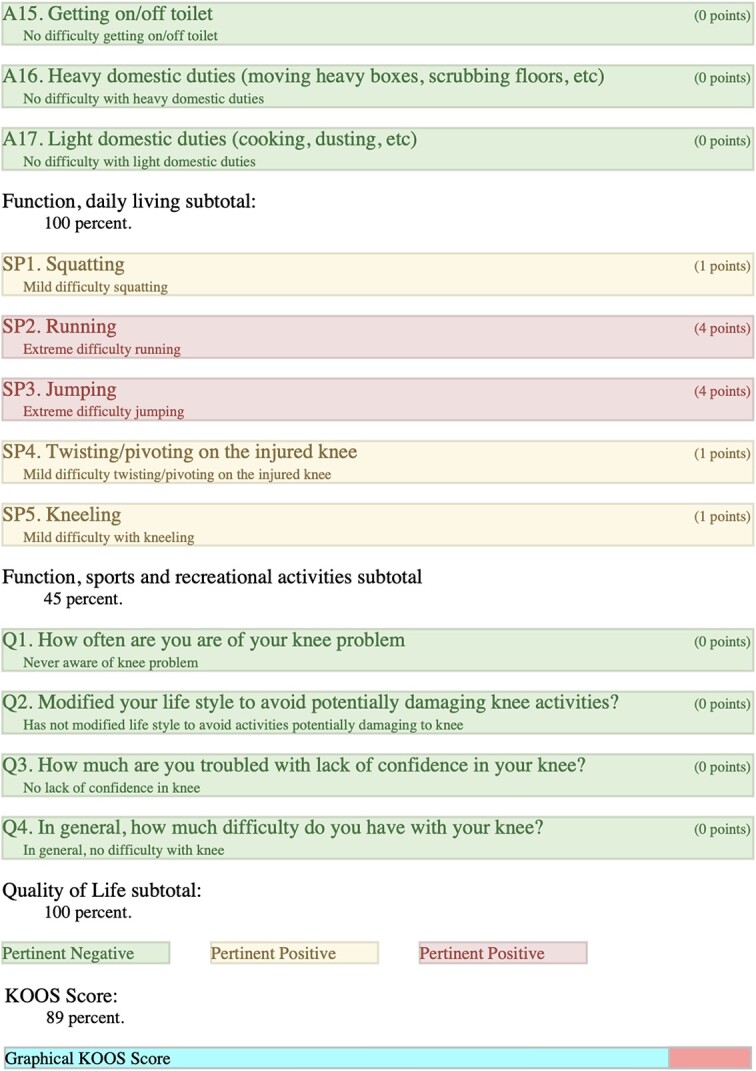

### Statistical comparison/analysis

**Figure 11:**
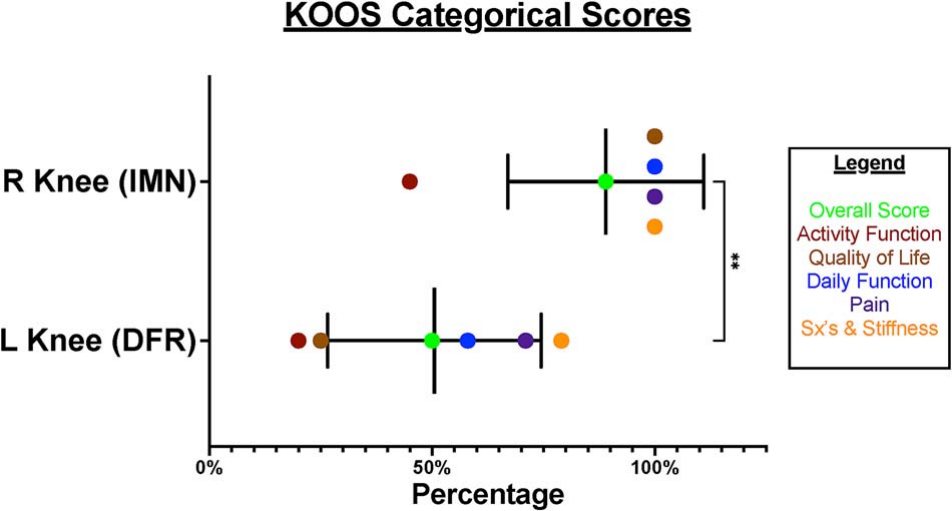
Graph, KOOS categorical scores.

KOOS individual scores were compared with a *P* value of (<0.0001), standard deviation of (1.248), R squared of (0.5562), and a 95% confidence interval of (−1.7700) to (−0.9919). KOOS categorial subtotals and overall percentages were compared with a *P* value of (0.0048), standard deviation of (19.91), R squared of (0.8222), and a 95% confidence interval of (17.92) to (59.08) (Fig. 11).

## Discussion

There is a growing interest in orthopedic medicine in comparing the efficacy of DFR versus ORIF in treating distal total knee periprosthetic femur fractures. There is a lack of comprehensive research analyzing the comparative outcomes of these two treatment options, and therefore, there is no consensus on what is optimal for this fracture type. This discussion briefly highlights the current literature that evaluates these two treatment methods and summarizes the outcomes observed in a unique patient case series who underwent bilateral total knee arthroplasties in 2010 with subsequent independent bilateral periprosthetic femur fractures (Table 5). She provided objective and subjective data on the efficacy of DFR versus ORIF in her specific circumstance.

**Table 5:** Current literature outline.

Citation #	Authors	Journal	Year	Patient #	Study	Complications	Study type
1	Brian D, MD, Daniel D. Bohl MD MPH, Mark S. Karadsheh MD, Scott M. Sporer MD MS, Richard A. Berger, MD, Brett R. Levine, MD, MS	The Journal of Arthroplasty	2020	72 with minimum 2-year follow-up	Compare ORIF to DFR	There was no appreciable difference in estimated blood loss (438 vs 363 mL). The mortality rates were high in both groups, with six ORIF patients (12%) and seven DFR patients (32%) dying within the initial 2 years of surgery. Infection rates were comparable for both methods.	Case study
2	John A. Ruder, MD, Gavin P. Hart, MD, Jeffrey S. Kneisl, MD, Bryan D. Springer, MD, Madhav A. Karunakar MD	The Journal of Arthroplasty	2017	58 patients	Review of distal femoral periprosthetic fractures treated with either ORIF or DFR	One patient had deep venous thrombosis in ORIF versus two in DFR. One year mortality rate was 20% in both methods. There was one malunion and one non-union incidence for ORIF but none for DFR. There were two incidences of wound complication requiring tissue coverage and one incidence for superficial infection in DFR but none for ORIF.	Retrospective review
3	David A. Quinzi, Gabriel Ramirez, Nathan B. Kaplan, Thomas G. Myers, Caroline P. Thirukumaran & Benjamin F. Ricciardi	Archives of Orthopaedic and Trauma Surgery	2021	353 DFR cases, 272 IMN cases, and 1205 ORIF cases	Systematic review and meta-analysis to evaluate complication and revision rates for periprosthetic distal femur fractures (PPDFF) treated with: (i) ORIF, (ii) retrograde IMN, and (iii) DFR.	DFR group had a higher incidence of deep infection relative to IMN and ORIF groups. Malunion rates were higher in IMN versus ORIF.	Systematic review
4	Caines, Andrew MD, Adamczyk, Andrew MD, MSc, FRCSC, Mahaffey, Ryan MD, FRCPC, Pickell, Michael MD, MSc, FRCSC	Journal of Orthopaedic Trauma	2023	39 patients	To determine the economic cost associated with the treatment of OTA/AO 33C fractures in patients older than 65 years of age using ORIF or DFR and to assess the perioperative outcomes.	There was one case of perioperative mortality in the DFR group (intraoperative arrest), as well as one case in the ORIF group (postoperative pneumonia). One-year mortality rate was 0 of 11 in the DFR group compared with 3 of 26 in the ORIF group which was not significant. Five patients (19%) in the ORIF group required reoperation, with four cases of reoperation for nonunion with one revision ORIF and three revisions to DFR.	Retrospective Cohort over a 10-year period
5	Hussain, Mohammed S. MD, Dailey, Steven K. MD, Avilucea, Frank R. MD	Journal of Orthopaedic Trauma	2018	9 patients	Weight-bearing after combined retrograde intramedullary nailing and ORIF of noncomminuted distal interprosthetic femur fractures	No postoperative infections, gross implant failures, screw pull out, implant loosening, pulmonary embolism, or deaths. One deep vein thrombosis was identified with a duplex and managed with a 3-month course of warfarin.	Comparative study
6	Hosam E. Matar, BSc (Hons), MSc (Res), FRCS (Tr&Orth), Benjamin V. Bloch, BSc (Hons), MBBS, FRCS (Tr & Orth), and Peter J. James, BMedSci, BMBS (Hons), DipBiomech, FRCS, FRCS (Tr&Orth)	Arthroplast Today	2021	30 patients	The aim of this study was to evaluate short- to medium-term outcomes of comminuted periprosthetic distal femoral fractures treated with DFR at a tertiary arthroplasty unit.	Two patients (2/27; 7.4%) developed complications; one patient required reoperation at 7 years for change of polyethylene which had dislocated after a fall. 1 reoperation for insert dislocation 1 patella dislocation	Retrospective consecutive study
7	Meng-Qiang Fan, Xiao-Lei Chen, Yong Huang, Jie-Feng Huang	Arthroplasty	2020	2 patients	Case review of two patients with periprosthetic proximal femur fracture variant and femoral stem destabilization.	N/A	Case report
8	Christ, Alexander B. MD, Chawla, Harshvardhan MD, Gausden, Elizabeth B. MD, Villa, Jordan C. MD, Wellman, David S. MD, Lorich, Dean G. MD, Helfet, David L. MD	Journal of Orthopaedic Trauma	2018	40 patients	Retrospective case review of 40 patients treated by 3 surgeons using ORIF	No deep infections. There were two nonunions that required revision osteosynthesis. There 3 for revision surgery.	Retrospective case series
9	Gerrard Gan, MRCS, Yee Hong Teo, FRCS, Ernest Beng Kee Kwek, FRCS	Clinics in Orthopedic Surgery	2018	15 patients	This is a retrospective study of 15 patients who underwent either tumor prosthesis revision or locking plate fixation for supracondylar femoral periprosthetic fractures from 2009 to 2014.	6 developed postoperative anemia that required two or more blood transfusions; 2 developed fast atrial fibrillation as a result of postoperative pain. 2 developed uncomplicated urinary tract infection as a result of immobility. One patient was found to have septic nonunion at 13 months from the fixation operation.	Retrospective study

A study by Darrith et al. involved analyzing 72 patients who suffered from a displaced periprosthetic DF fracture and had a minimum follow-up period of 2 years. Of these patients, 50 received ORIF treatment, while 22 received DFR treatment. The study assessed the outcomes using multivariate regression analysis, which included Knee Society Scores, infection rates, revision incidence, and mortality. The Knee Society Functional Score preferred ORIF, but the total revision incidence was higher in the ORIF group. Both treatment modalities had a high mortality rate and a significant risk of reoperation [8]. According to a study by Caines et al., patients with a DFR procedure showed better postoperative mobility than those who underwent ORIF. Although patients with ORIF had a shorter subacute length of stay, the study found that a higher proportion of DFR patients could mobilize postoperatively compared to ORIF patients, even though ORIF patients had a shorter subacute length of stay [ 9].

When comparing the two knees and, in effect, comparing the two surgical interventions, we considered a comprehensive range of factors. We examined the patient’s KOOS surveys, ROM differences, pain scores, balance, strength, subjective functionality, and self-reported quality of life impact after both periprosthetic surgeries. Our patient experienced their first periprosthetic femur fracture on the left side in 2020, which was subsequently treated with a revision total knee arthroplasty. The patient reported that she faced more difficulties in recovering from her fracture on the left side, resulting in higher pain levels, decreased ability to move around, lower quality of life, and a sensation of being “off balance” (KOOS overall score of 50%). In 2022, the patient experienced a second periprosthetic femur fracture, this time on the right side. The patient underwent a retrograde IMN fixation, and the patient reported no lasting impact or severe pain in her right knee after the surgery, as indicated by a KOOS overall score of 89%. Furthermore, the patient reported minimal disruption to her quality of life due to her right knee.

The evidence from this patient alone may suggest that a retrograde IMN fixation is a better long-term procedure than revision total knee replacement for patients with periprosthetic fractures. The authors hypothesize that using a retrograde nail, which provides increased stability and is considered a less invasive procedure, maybe the main contributing factor to the more favorable long-term outcome associated with its use. Compared to DFR, which inherently causes more instability and is more invasive, it may have resulted in a poorer outcome.

Acknowledging that the report is subject to certain inherent limitations is imperative. The patient had similar but objectively distinct periprosthetic fracture patterns in each knee; she had an intrinsic periprosthetic fracture history before her right knee fracture; moreover, the treatment process involved two surgeons who made autonomous decisions without any intercommunication. Nonetheless, this limitation can also be seen as beneficial, as the surgical interventions were independently evaluated and treated based on each surgeon’s methodology. Lastly, the report is based on a single patient case series, which makes it difficult to generalize the findings to a broader population and conclusions made in this paper are based solely on a sample size of one.

## Conclusion

Based on a single patient and this report’s data alone, performing an IMN for DF periprosthetic fractures should be considered the first choice based on this patient’s improved pain, functionality, and overall satisfaction, even if anatomic alignment is difficult. The importance of considering individual patient factors when deciding on the most effective treatment option must be balanced, and our patient’s feedback highlights this. Her positive results emphasize the significance of using this case series to develop a treatment plan for patients with similar fractures. Given the complexity of the issue and the study’s implications, we strongly recommend more clinical and multicenter trials to determine the optimal treatment for this type of fracture. The comparison of these two treatments requires additional randomized control trials to provide comprehensive data-driven conclusions, and until further research is conducted, the limited data available hinder making definitive judgments.
